# Optimization AlGaN/GaN HEMT with Field Plate Structures

**DOI:** 10.3390/mi13050702

**Published:** 2022-04-29

**Authors:** Ningping Shi, Kejia Wang, Bing Zhou, Jiafu Weng, Zhiyuan Cheng

**Affiliations:** 1 School of Materials Science and Engineering, Key Laboratory of New Processing Technology for Nonferrous Metals and Materials of Ministry of Education, Collaborative Innovation Center for Exploration of Nonferrous Metal Deposits and Efficient Utilization of Resources, Guilin University of Technology, Guilin 541004, China; shiningping1997@gmail.com (N.S.); 13695032900@163.com (J.W.); 2 School of Micro-Nano Electronics, Zhejiang University, Hangzhou 310027, China; zycheng@zju.edu.cn; 3 Ningbo Haitechuang Electric Control Co., Ltd., Ningbo 315000, China

**Keywords:** field plate, GaN HEMT, TCAD simulation, breakdown voltage, FOM

## Abstract

AlGaN/GaN HEMTs with several different designs of field plate structure are studied for device optimization purposes. To increase device breakdown voltage, optimal dimensions of field plates were first investigated using Silvaco TCAD software, and the electrical characteristics of the devices are analyzed. Several devices were designed and fabricated based on the simulation results. It has been confirmed that the gate-source composite field plate (SG-FP) has a higher breakdown voltage than other types of field plate structures, with FOM reaches 504 MW/cm^−2^, showing that the device with SG-FP structure outperforms the other three structures. The experiment and simulation verify that the gate-source composite field plate optimizes FOM by increasing the breakdown voltage and reducing the intrinsic on-resistance so that the device has better electrical performance and a wider application range.

## 1. Introduction

GaN is the most representative wide band semiconductor material, and the GaN material system has the best theoretical electro-optical and photoelectric conversion efficiency to date, making it a viable alternative for high-temperature, high-frequency, high-power microwave devices. GaN has a wide bandgap of 3.4 eV, a critical breakdown electric field of 3.5 MV/cm, a high saturated electron drift velocity of 2.5 × 10^7^ cm/s, and a small dielectric constant of 9.8, when compared to SiC [1,2]. Johnson’s figure of merit (FOM) can be used to evaluate the comprehensive performance of power electronic devices. The formula is FOM = V_BV_^2^/R_on.sp_. V_BV_ is the breakdown voltage and R_on.sp_ is the intrinsic on-resistance [3,4]. With the development of large-size and high-quality Si-based GaN technology, GaN materials have great application potential in the field of power electronics. AlGaN/GaN HEMT devices can achieve lower on-resistance than other materials, which reduces the open-state losses and improves the conversion efficiency of the system [5,6].

The uses of a field plate structure in GaN devices have been shown in previous papers to improve breakdown voltage and suppress current collapse. Due to the current collapse phenomenon in high-voltage GaN devices, the on-resistance increases with increasing applied voltage [7]. By optimizing the gate field plate length (L_GFP_), source field plate length (L_SFP_), and passivation layer thickness, the electric field distribution between the drain and the gate is more uniform, and the voltage resistance of the device is improved. In 2019, Hemanth [8] et al. simulated gate field plates with different stresses, and the breakdown voltage was up to 1574 V, and the Dynamic Ron ratio was 4.1 Ω. In 2020, Godfrey [9] et al. calculated the maximum withstand voltage of 1310 V by simulating different lengths of source field plates. In 2010, Saito [7] et al. fabricated high-voltage GaN HEMTs with various field plate structures. With the intrinsic on-resistance R_on.sp_ of 3.9 mΩ·cm^2^ and the breakdown voltage of 680 V, the gate-source composite field plate (SG-FP) was the best. In 2014, Wang [10] et al. simulated three kinds of dual-field plate structures, the gate-source composite field plate structure, discontinuous gate field plate and source field plate structure, and gate-source composite discontinuous field plate structure. The gate-source composite field plate structure can improve the peak electric field at the edge of the gate field plate, and generate a new peak at the edge of the source field plate, with the breakdown voltage reaching 1365 V. In 2020, Qiaoyu Hu [11] et al. demonstrated the gate-source composite field plate (SG-FP) has a breakdown voltage of over 600 V, and the dynamic R_on_ is 50% higher than the static R_on_.

In this paper, AlGaN/GaN HEMT devices with different field plate structures are investigated. The tape-out results demonstrate that the gate-source composite field plate can improve the peak electric field and increase the breakdown voltage. At the same time, the field plate length of the device is optimized by simulation, and the breakdown voltage and FOM of the device are further improved.

## 2. Device Design and Simulation Model

**Figure 1:**
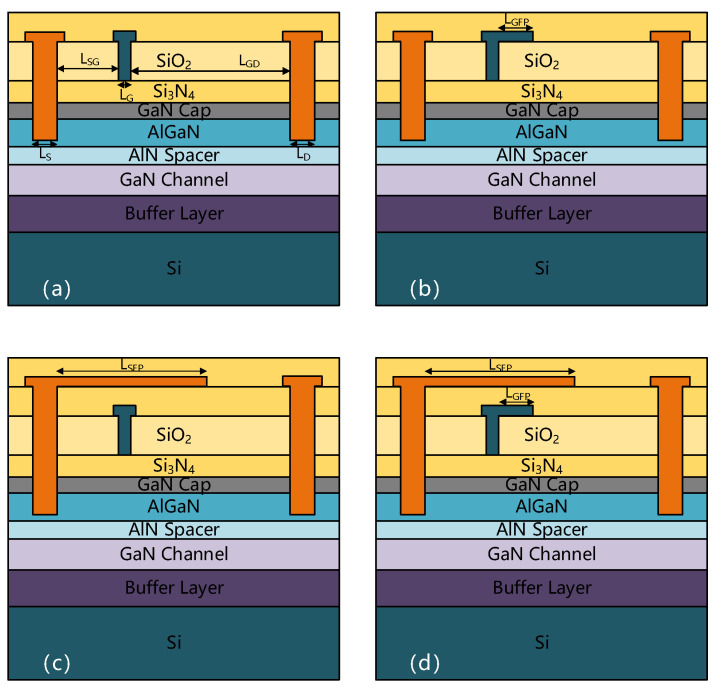
Schematic diagram of AlGaN/GaN HEMTs device structure and its field plate variation. (**a**) No FP. (**b**) G-FP. (**c**) S-FP. (**d**) SG-FP.

The GaN HEMT structures are shown in Figure 1, with the structure of the fieldless plate (no FP), the gate field plate (G-FP), the source field plate (S-FP), and the gate-source composite field plate (SG-FP) with gate and source field plate. From bottom to top, Si substrate, 3 μm buffer layer, 300 nm GaN channel, 1 nm AlN spacer, 25 nm AlGaN barrier layer (Al content is 20%), 2 nm GaN cap layer, 20 nm Si_3_N_4_, 200 nm SiO_2_ and 300 nm Si_3_N_4_ are included.

**Figure 2:**
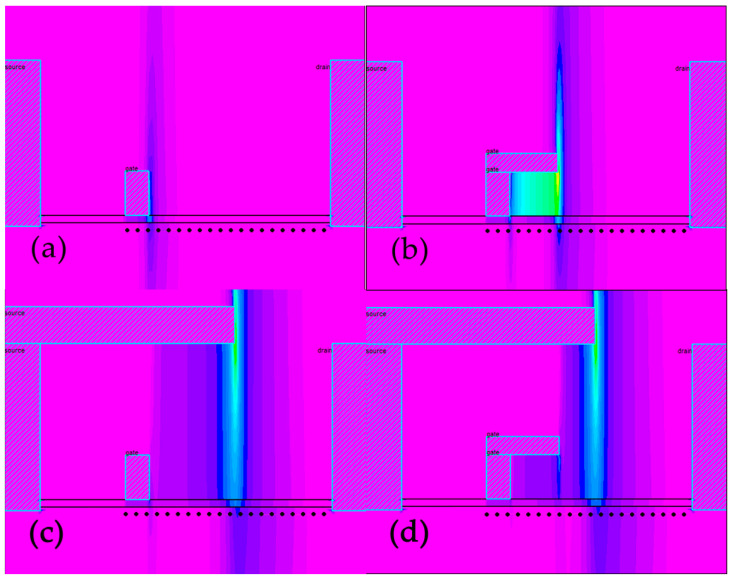
Electric field simulation of different types of AlGaN/GaN HEMT field plates. (**a**) No FP. (**b**) G-FP. (**c**) S-FP. (**d**) SG-FP.

In order to improve the breakdown voltage of AlGaN/GaN HEMT, the field plate of AlGaN/GaN HEMT is simulated by Silvaco TCAD software. The simulation structure is shown in Figure 2. In the parameter setting, the gate length L_G_ = 2 μm, the source and the drain length L_S_ = L_D_ = 3 μm, the distance between the source and gate L_SG_ = 7 μm, and the distance between the drain and gate L_GD_ = 15 μm. The physical models used in the simulation include the field-dependent mobility (fldmob), Fermi-Dirac statistics (fermi), domain-related mobility (print), and SRH recombination (SRH). For the breakdown simulation, the collision ionization model (selb) is used. The formula of collision ionization model is α_0_·exp (−E_c_/E), α_0_ is the ionization coefficient of 2.9 × 10^8^ cm^−1^, E_c_ is breakdown field of 3.4 × 10^7^ V/cm. Dense mesh distribution at gate edge and field plate edge. The trap density at the interface between Si_3_N_4_ and AlGaN is 1 × 10^−7^ cm^−2^. AlGaN and GaN doped 1 × 10^18^ cm^−3^ concentration. The material parameters needed in the simulation are shown in Table 1.

**Figure 3:**
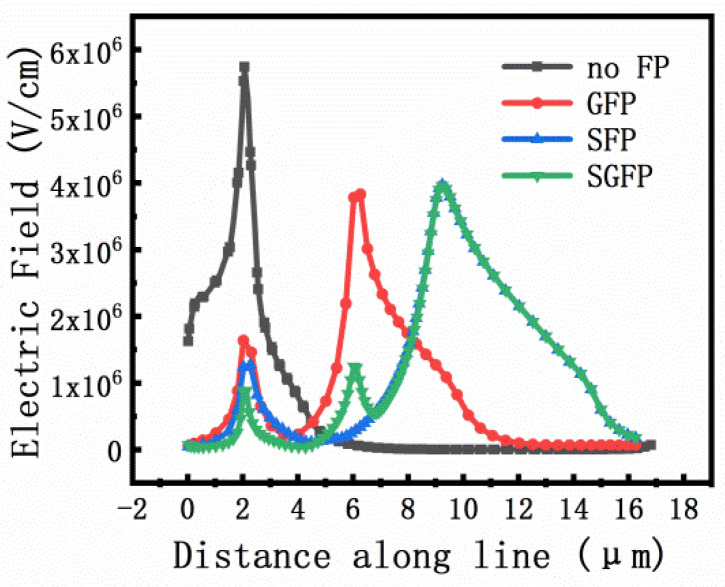
Simulated electric field strength of different field plate types.

In HEMT devices, the electric field between the gate and drain is not uniformly distributed. Under the high potential difference between the external gate and drain electrode, there is a large electric field peak near the edge of the drain electrode. Moreover, the peak electric field increases with the increase in the potential difference between the gate and drain electrodes, which is the primary cause of device breakdown [13]. The electric field distribution of different types of field plates is measured by cutting along the black line in Figure 2 as shown in Figure 3, which indicates the mechanism of the field plate structure as shown.

**Figure 4:**
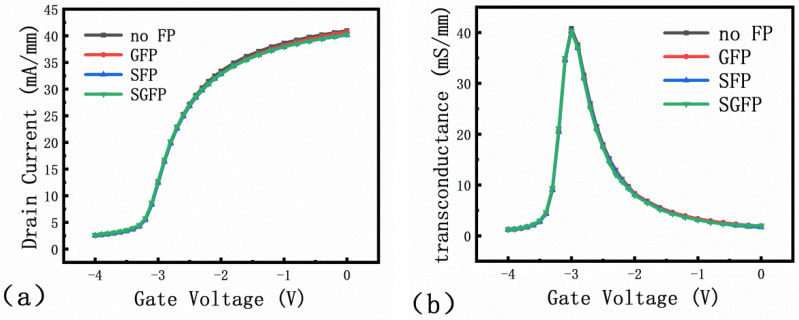
Simulation of (**a**) transfer characteristic curves and (**b**) transconductance for different field plate types.

According to the actual process flow, the passivation layer should be SiO_2_ and Si_3_N_4_ [14], and in the simulation the device structure was simplified and set Si_3_N_4_ as the passivation layer. Figure 4a shows the transfer characteristics of the devices with different field plate structures and the threshold voltage (V_th_) is −3.37 V. Since all the devices investigated in this study are depletion types, the V_th_ is negative. As shown in Figure 4, the threshold voltage is the same for devices with different types of field plate structures [15]. The transconductance (g_m_) indicates the ability of the gate to regulate the channel current and also determines the switching speed of the device. The larger the transconductance, the faster the device switches [16]. In Figure 4b, the transconductance is calculated by differentiating the results from the transfer characteristic curve, and the transconductance g_m_ = 40.2 mS/mm, which is essentially the same. It shows that the threshold voltage and transconductance are unaffected by the field plate structure.

**Figure 5:**
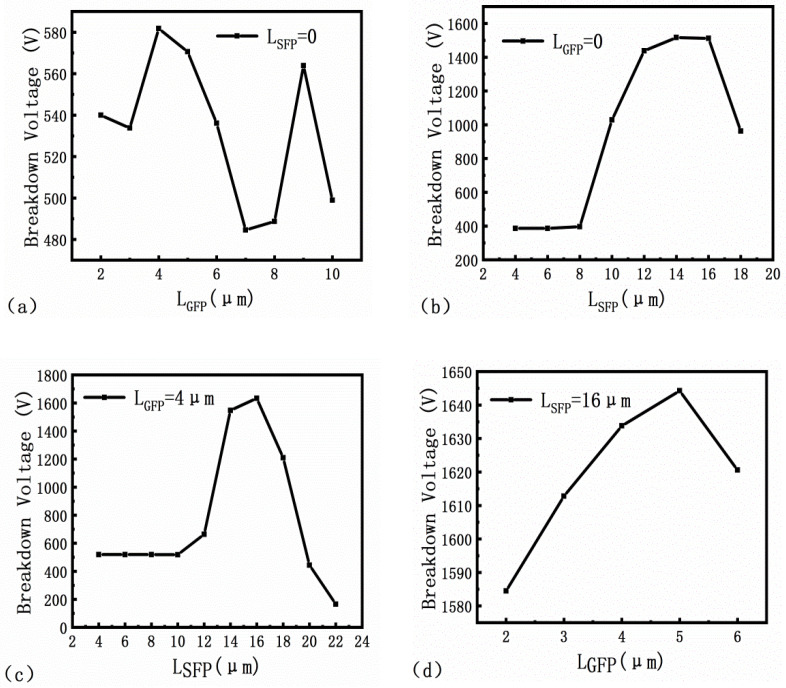
Variation of breakdown voltage with field plate length (**a**) single G-FP; (**b**) single S-FP; (**c**) SG-FP with L_GFP_ = 4 μm; (**d**) SG-FP with L_SFP_ = 16 μm.

Figure 5a shows that the breakdown voltage of the single G-FP AlGaN/GaN HEMT device varies with the length of the gate field plate. The reverse IV characteristic curve is obtained by applying 5.23 V to the gate and scanning the source-drain voltage from 0 V to 1200 V. The breakdown voltage is the corresponding drain voltage when the device is burned, which is an irreversible process. Once the device reaches this breakdown point, the leakage current will increase significantly and the device will be permanently destroyed [17]. When the gate field plate length (L_GFP_) increases from 2 μm to 10 μm, the breakdown voltage reaches the maximum of 581.8 V at L_GFP_ = 4 μm, and suddenly increases to 563.9 V at L_GFP_ = 9 μm. The breakdown voltage decreases as the L_GFP_ increases from 4 μm to 7 μm. Because the peak value of the electric field at the edge of the gate field plate continues to shift to the drain, the influence on the electric field intensity at the edge of the gate is weakened. Since the on-resistance R_on_ increases as the gate-drain spacing increases, the maximum L_GFP_ should be half of the gate-drain spacing, which is 7.5 μm [7]. As a result, L_GFP_ = 4 μm is the length of the chosen gate field plate.

The breakdown voltage of the single S-FP is shown in Figure 5b. The length of the source field plate increases to 8 μm when the passivation layer thickness remains constant, while the breakdown voltage remains constant. This is because the distance between the gate source plus the gate distance of 9 μm is not exceeded by the source field plate [18]. The breakdown voltage increases continuously as the length of the source field plate increases from 8 μm to 16 μm. The maximum breakdown voltage is 1512.6 V at 16 μm. Then, with the increase in the length of the field plate, the breakdown voltage begins to decrease. The gate field plate increases the gate-drain feedback capacitance, which has a negative impact on the power gain. The source field plate is made on a passivation layer thicker than the gate. It can reduce the strong electric field of the gate-drain side and increase the capacitance of the source-drain, so the negative effect is less than that of the gate field plate [19].

Figure 5c,d simulates the breakdown voltage of the gate-source composite field plate structure with different field plate lengths. Figure 5c shows the simulation result with different L_SFP_s when L_GFP_ = 4 μm, the breakdown voltage can reach up to 1633.5 V when L_SFP_ = 16 μm. Figure 5d shows that when L_SFP_ = 16 μm is used to simulate different L_GFP_s, the breakdown voltage can reach 1644.3 V when L_GFP_ = 5 μm.

**Figure 6:**
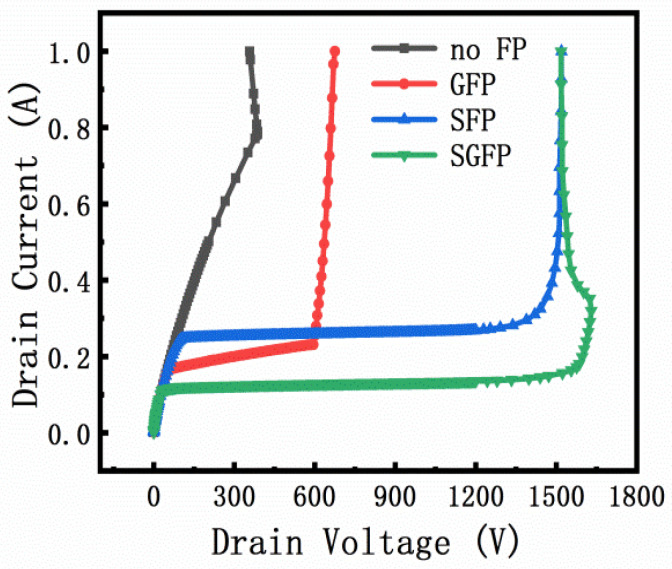
Breakdown voltage characteristics for the design of no FP, G-FP, S-FP and SG-FP, for L_GFP_ = 4 μm and L_SFP_ = 16 μm.

The breakdown voltage of the field plate type designed in this paper is shown in Figure 6, as 387 V, 1186.2 V, 1512.6 V, and 1633.5 V, respectively. The size of the SG-FP is L_GFP_ = 4 μm and L_SFP_ = 16 μm. The breakdown voltages of the single G-FP and single S-FP are 3.1 and 3.9 times higher than those of the no FP, respectively. On the other hand, SG-FP has a higher breakdown voltage, 4.2 times higher than that of the no FP.

From the perspective of optimizing the device structure, the above simulation compares the breakdown characteristics of gate field plate length, source field plate length, and gate-source composite field plate length to determine the device’s optimal size. The field plate structure reduces the peak electric field at the gate edge, thereby increasing the breakdown voltage, weakening the strong field electron effect, suppressing the current collapse, and increasing the output power.

## 3. Experimental Results and Tests

**Figure 7:**
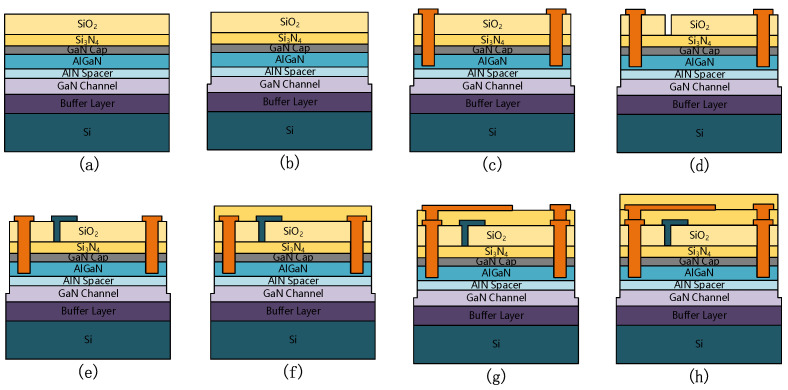
Fabrication flow chart of AlGaN/GaN HEMT device. (**a**), passivation: SiO_2_ dep; (**b**) mesa etch; (**c**) ohmic contact; (**d**) gate trench etching; (**e**) gate metal dep; (**f**) passivation: Si_3_N_4_ dep; (**g**) ohmic metal dep; (**h**) surface passivation: Si_3_N_4_ dep.

**Figure 8:**
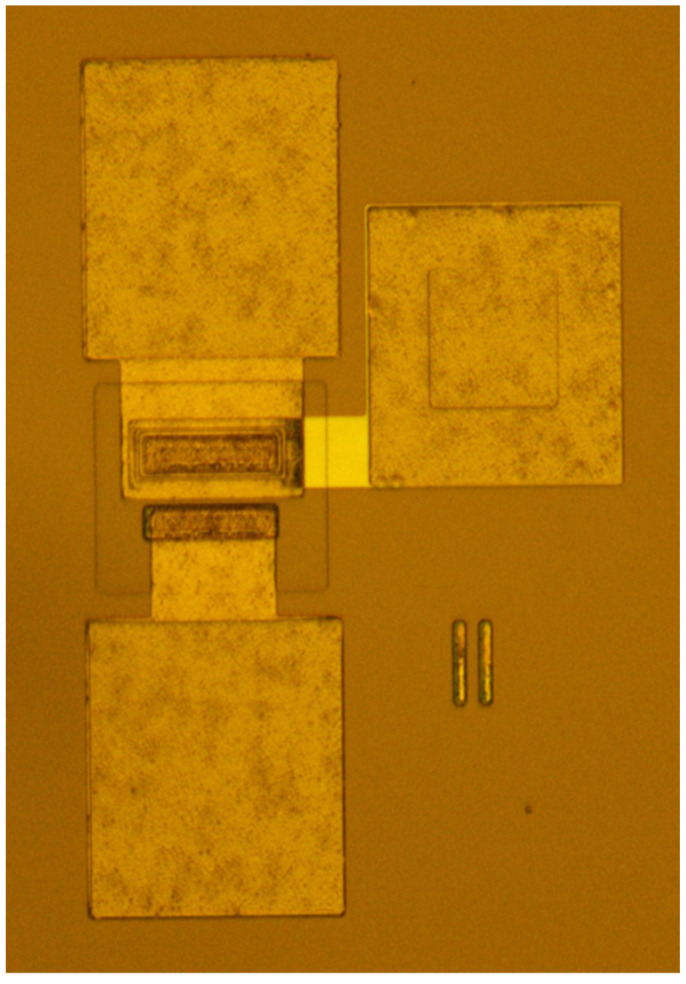
AlGaN/GaN HEMT device optical micrograph.

The epitaxial wafer used in this study is consistent with the simulation structure, consisting of 2 nm GaN cap layer, 25 nm AlGaN barrier layer (Al content is 20%), 1 nm AlN spacer, 300 nm GaN channel and 3 μm buffer layer. The fabrication process of HEMT is shown in Figure 7: a passivation: SiO_2_ dep; b. mesa etch; c. ohmic contact; d. gate trench etching; e gate metal dep; f. passivation: Si_3_N_4_ dep; g. ohmic metal dep; h. surface passivation: Si_3_N_4_ dep. The thickness of SiO_2_ is 200 nm and the thickness of Si_3_N_4_ is 300 nm. The ohmic metal material is Ti/Al/Ni/Au. The gate metal material is Ti/Au. Four AlGaN/GaN HEMT devices with different field plate structures were designed and fabricated to study the breakdown voltage and FOM of the device. Where L_GFP_ of the single G-FP is 4 μm; L_SFP_ of single S-FP is 16 μm; and L_GFP_ = 4 μm, L_SFP_ = 16 μm for SG-FP. The device optical micrograph image is as shown in Figure 8.

**Figure 9:**
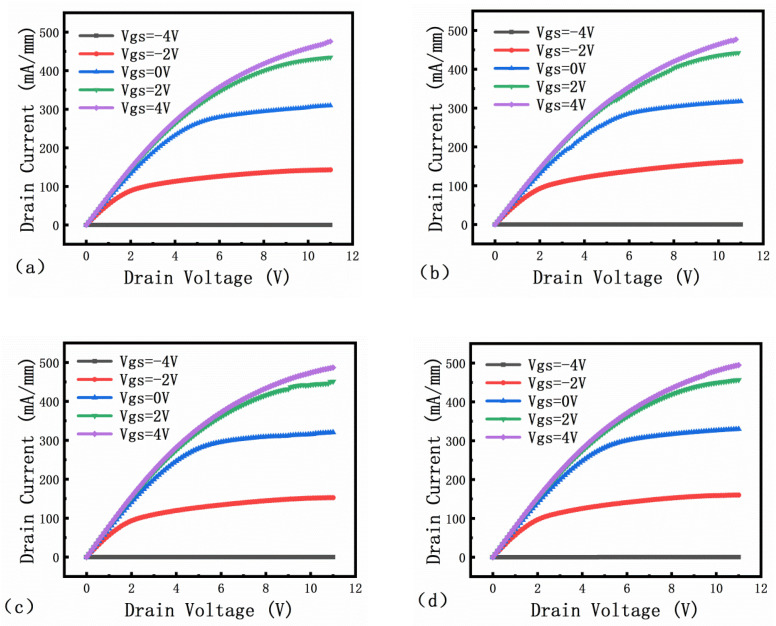
I–V characteristics of (**a**) no FP, (**b**) G-FP, (**c**) S-FP, (**d**) SG-FP.

Figure 9 shows the output characteristics of four different field plate structures. The source-drain voltage (Vds) varies from 0 V to 11 V, and the gate voltage (VG) varies from −4 V to 4 V. From the output curve, it can be seen that when VG = 4 V, the maximum saturation current (Ids) of no FP, G-FP, S-FP, and SG-FP are 476 mA/mm, 477.1 mA/mm, 487 mA/mm, and 494.7 mA/mm, respectively. The difference is small, which is related to the density of two-dimensional electron gas [16].

**Figure 10:**
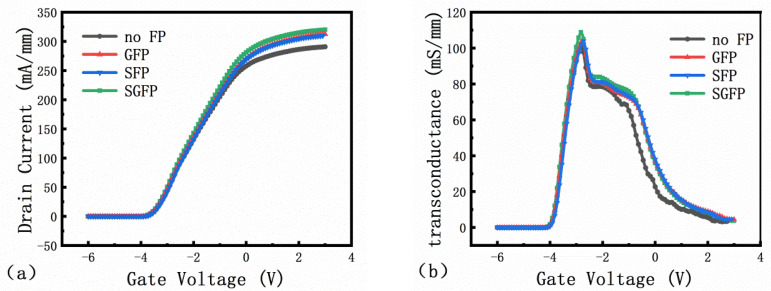
Vds = 6 V (**a**) Transfer characteristic curve; (**b**) Transconductance.

Figure 10a shows the transfer curves of the devices with different field plate structures. When the source-drain voltage (Vds) is 6 V, the gate voltage is scanned from −6 V to 3 V. The threshold voltages (V_th_) remain nearly constant as V_th_ = −3.9 V. Figure 10b shows the transconductance (g_m_) of the device as 100.7 mS/mm@VG = −2.8 V, 102 mS/mm@VG = −2.8 V, 103.2 mS/mm@VG = −2.8 V, and 108.9 mS/mm@VG = −2.8 V, respectively. R_on_ and R_on.sp_ can be calculated from the formula R_on_ = 1/g_m_ [20].

**Figure 11:**
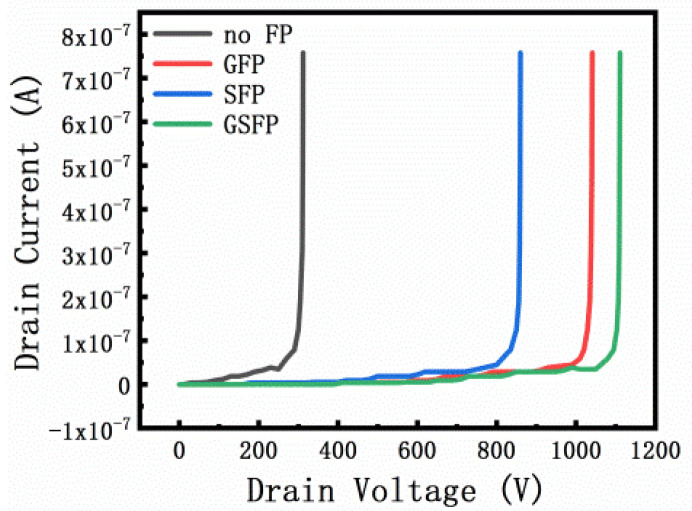
Breakdown voltage of devices with different field plate types.

As shown in Figure 11, the breakdown voltage characteristics of AlGaN/GaN HEMT devices with different field plate structures are significantly improved. By selecting the optimal field plate structure and field plate length, the breakdown voltage of the device increases from 312 V of the no FP to 860 V of the G-FP, 1041 V of the S-FP, and 1118 V of the SG-FP. In addition, the leakage of the device at high voltage is reduced, allowing the field plate structure to modulate the electric field [9]. From the previous simulation results, we understand the impact of the field plate structure and size on the breakdown voltage of the device, and obtain the optimal size structure. By using the optimal size structure, it can be found that the breakdown voltage of the S-FP and the SG-FP is smaller than that of the simulated device. On the contrary, in the G-FP, the actual breakdown voltage is greater than the simulated breakdown voltage. When simulating G-FP, the electric field is concentrated below the gate field plate. From Figure 2b, it can be seen that the electric field intensity is large and that the peak value of the electric field is high, which leads to the breakdown of the simulation model in advance. SG-FP also has a gate field plate structure. However, under the action of the source field plate, the electric field peak shifts towards the drain, making the electric field distribution under the gate field plate more uniform. The simulation is carried out under ideal conditions, and there are defects, impurities, temperature rise, and other factors in the actual manufacturing. Rough model and grid distribution issues also result in a considerable gap between theoretical and actual values.

The calculation of FOM uses the following formula: FOM = V_BV_^2^/R_on.sp_. As the formula shows, the FOM is increased by increasing the breakdown voltage or reducing the intrinsic on-resistance. In summary, the device has a maximum FOM of 504 MW/cm^2^ with the SG-FP structure. Table 2 lists the specific experimental data.

## 4. Conclusions

In this paper, 4 μm gate field plate length and 16 μm source field plate length are obtained as the optimized size of the device with Silvaco simulation for different gate field plate lengths and source field plate lengths. Four devices with different field plate types are taped out, and the characterization results are consistent with the simulation. The field plate structure affects the breakdown voltage and electric field intensity distribution while having a minor impact on the device’s DC characteristics. The breakdown voltage of the SG-FP is about 3.6 times that of the no FP. Simultaneously, it is shown that the SG-FP has a more uniform electric field, a higher breakdown voltage, and a higher FOM than the single-gate and single-source field plate structures. The AlGaN/GaN HEMT device with the gate-source composite field plate with a high breakdown voltage as 1118 V and a low intrinsic on-resistance as 2.48 mΩ·cm^2^ are obtained. Selecting the optimal field plate length under the optimal field plate structure can maximize the device’s breakdown voltage. The research results show that the gate-source composite field plate structure has a good prospect. Simulation before tape out can save on cost.

## Figures and Tables

**Table 1:** Parameters for simulation [12].

Parameters	GaN	AlGaN
Eg300 (eV)	3.4	3.96
Align	0.8	0.8
Permittivity	9.5	9.5
Saturation Velocity (V_satn_)	1.9 × 10^7^ cm/s	1.1 × 10^7^ cm/s
Electron Low Field Mobility (Mun)	900 cm^2^/Vs	600 cm^2^/Vs
Hole Low Field Mobility (Mup)	10 cm^2^/Vs	10 cm^2^/Vs
300K conduction band state density (Nc300)	2.24 × 10^18^ cm^3^	2.07 × 10^18^ cm^3^
300K valance band state density (Nv300)	1.16 × 10^19^ cm^3^	1.16 × 10^19^ cm^3^

**Table 2:** Static parameters.

	No FP	G-FP	S-FP	SG-FP
Ids (mA/mm)	476	477.1	487	494.7
Vth (V)	−3.9	−3.9	−3.9	−3.9
gm(mS/mm)	100.7	102	103.2	108.9
V_BV_ (V)	312	860	1041	1118
R_on_ (Ω)	9.9	9.8	9.7	9.2
R_on.sp_ (mΩ•cm^2^)	2.67	2.65	2.62	2.48
FOM (MW•cm^2^)	36.5	279.1	413.6	504

## Data Availability

Not applicable.
